# Predictors of malaria vaccine uptake among children 6–24 months in the Kassena Nankana Municipality in the Upper East Region of Ghana

**DOI:** 10.1186/s12936-022-04378-1

**Published:** 2022-11-16

**Authors:** Dominic Yeboah, Joseph Owusu-Marfo, Yaa Nyarko Agyeman

**Affiliations:** 1 Regional Disease Control Unit, Ghana Health Services, Bolgatanga, Ghana; 2 Department of Epidemiology, Biostatistics and Disease Control, School of Public Health, University for Development Studies (UDS), Tamale, Norther Region Ghana; 3 Department of Population and Reproductive Health, School of Public Health, University for Development Studies (UDS), Tamale, Norther Region Ghana

**Keywords:** RTS,S vaccine, Predictors, Vaccine uptake, Malaria, Ghana

## Abstract

**Background:**

The Malaria Vaccine Implementation Programme (MVIP) coordinates the routine implementation of the RTS,S vaccine pilot in strategically selected locations in Malawi, Kenya, and Ghana. The pilot programme thoroughly assesses the programmatic feasibility of administering the four doses of the RTS,S vaccine. It will also assess the impact on malaria morbidity and mortality, as well as monitor and detect the vaccine's safety for routine usage. The malaria vaccine was introduced into Ghana's routine vaccination programme in May 2019 in seven regions, comprising 42 districts, including Kassena Nankana Municipal in the Upper East region of Ghana. Therefore, this study seeks to assess the predictors of the malaria vaccine uptake in children 6 to 24 months in the Kassena Nankana Municipal in Ghana.

**Methods:**

The survey used a cross-sectional study design and included 422 mothers/caregivers with children aged 6 to 24 months from the Kassena Nankana Municipality. WHO cluster survey questionnaire was altered for use in data gathering with caregivers as respondents. The Statistical Package for the Social Sciences (SPSS) version 25.0 (for descriptive statistics) and Stata version 13 (for calculating odds ratios) were used to analyse the data.

**Results:**

The findings depict that, the mean age of respondents for the study was 27 ± 5 years and average age of children was 15 ± 8 months. The study found that coverage uptake was high (94%). Chi-square and odds ratios testing revealed statistically significant associations between health service factors and vaccine uptake: education on malaria vaccine cOR(Cl); 9.69(3.496–25.425), (P < 0.001), giving caregivers the option to accept malaria vaccine cOR(Cl); 7.04 (2.759–17.476), (P < 0.001). Confidence in the efficiency of the vaccination was found to have a statistically significant association with malaria vaccine uptake (P < 0.005) and (p < 0.001) for ‘somewhat confidence’ and ‘not confidence at all’, respectively. Attitude of health workers was found to be significant predictor of malaria vaccine uptake (P < 0.003).

**Conclusion:**

Malaria vaccine uptake was high among the study population in the municipality; however, dose four uptake coverage by age two was low. This indicates that mothers/caregivers did not understand the notion of immunization throughout the second year of life. As a result, it is recommended that the municipality raise awareness about immunization services among mothers/caregivers beyond year one in order to improve performance and reduce the risk of disease outbreaks in the municipality.

## Background

One of the vector-borne protozoan disease is malaria caused by *Plasmodium* species which is spread when a female *Anopheles* mosquito bites susceptible host. The disease remains one of the public health importance outcomes in developing countries [1]. Nevertheless, the disease is preventable and curable, it was documented in 2015 that about 212,000,000 new cases with 429,000 deaths was recorded globally, of which ninety percent of the cases and ninety-two percent of the deaths happened in African. Additionally, 219,000,000 new cases of malaria happened in 2017 of which 435,000 deaths were recorded globally and as usual, the most affected were children and pregnant women [2].

In Ghana malaria accounts for about 17.6 percent of the general out-patient department attendance, 13.7 percent of ward admissions, and 3.4 percent of total maternal deaths [3].

There is demonstration of re-emergence of malaria in most places that successfully reduce the disease burden and were free from malaria. This development characterizes main risk for control and prevention of malaria, signifying the importance for new approaches to implement the control and preventive strategies and interventions [4].

From the year 2000, efforts are being intensified to fight against the prevalence of malaria through varied concerted campaigns such as the “Roll Back Malaria” campaign, which help to drastically reduce the impact of the disease. These programmes led to first-time high intervention performances and scaling up of effective managements and treatments across Africa. The new goals for World Health Organization (WHO) for the global lessening of malaria incidence and mortality rates by a minimum of ninety percent by the year 2030 and the elimination of malaria in not less than 35 endemic countries by 2030 [5].

Momentous effort has been made globally with much progress with the aim of reducing the malaria prevalence and burden. This accomplishment is basically due to the interventions to protect the susceptible hosts by providing and promoting the use of long-lasting insecticidal nets (LLINs), seasonal in-door residual spraying in communities and households, and artemisinin-based combination therapy for the management and treatment of malaria infections [6]. The blend of these interventions has contributed to about 40% reduction in malaria incidence and a 50% decrease in infections due to *Plasmodium falciparum* parasites.

Even though the level of reduction of the malaria infection appeared convincing, the figures were still high and fall short of the 75% target set by the WHO to reduce malaria burden by 2015. To be able to achieve the set target in reducing the prevalence, more efforts, strategies and interventions are paramount, especially effective vaccines in high prevalent places [6]. Vaccines have proved to be most cost effective and efficient health interventions for the general public with the achievement of morbidities and mortalities prevention in most developing countries [7]. Nonetheless, in malaria prevention, vaccine is not the sole significant matter, but the effectiveness of the vaccine, the burden of the disease on the population, and the cost implications arising as a result of the introduction of the vaccines are among the common concerns [7]. Expanded Programme on Immunization (EPI) is responsible for vaccines and vaccination to control, eliminate and eradicate vaccine preventable diseases (VPDs). Having strong immunization systems to deliver vaccines to those who need them most play a significant role in achieving the health, equity and economic objectives of several global development goals. These include the 2030 Sustainable Development Goals (SDGs), the 2011–2020 Decade of Vaccines, the 2030 Universal Health Coverage (UHC) agenda, the 2011–2020 Global Vaccine Action Plan (GVAP), the Global Routine Immunization Strategy and Plan (GRISP), and the Regional Strategic Plan for Immunization 2014–2020 [8].

Malaria vaccine development is predicted to provide a low-cost intervention to contribute to the reduction of malaria episodes. The milestones regarding the development of malaria vaccine has hastened in the recent times with increased research motivating the unearthing of new vaccines and vaccine expertise, and many vaccine candidates are being moved through the vaccine development pipeline [9]. Statement from the WHO, indicates that the RTS,S vaccine is so far the most progressive vaccine candidate in the vaccine development trail, which is to serve as a complementary malaria control strategy that might possibly be added to the already existing interventions and not to replace the main preventive and treatment interventions [10].

According to the WHO, cost-effective malaria vaccine with high efficacy will suppress morbidities and mortalities and contributes to malaria control strategies that is needed, especially in most endemic places where health service and control strategies might be difficult to sustain. The RTS,S vaccine is the first malaria vaccine licensed for use and which is an indication of an important step headed for malaria control and prevention. On the other hand, if the RTS,S is not as effective as expected, it will create challenges, especially to evaluate the efficacy of the malaria vaccines [1]. The WHO has indicated that a whole sporozoite vaccination method has shown hopeful outcomes, encouraging immunity in small number trials in adults, but may not attain strong protection in malaria endemic population densities [1]. “Vaccines targeting both the preerythrocytic and the erythrocyte-invasive form of the parasite (merozoites) may repeal leap forward infections by neutralizing merozoites developing from infected hepatocytes, while vaccines targeting the sexual stages strive to interruption the transmission cycle.” Moving forward, multiple vaccines could be the next step toward malaria prevention [1].

With the introduction of the RTS,S vaccine as part of the control strategies in sub-Saharan Africa, there would be a great impact on healthcare delivery in the sub-region. RTS,S is the first malaria vaccine and it is now being piloted and implemented in three African countries, which is Ghana, Kenya and Malawi to inform policy direction on the scaling up and broader use of the vaccine [6]. In order to ensure successful malaria vaccine combination into the already existing malaria intervention programmes, the interaction and relationship between Expanded Programme on Immunization and communities must be made unequivocal [6]. Communities’ socio-cultural values, religion, believes and particular unique characteristics must be considered so as to ensure acceptance to immunization services and these are key strategies that address the human realities in vaccine trials and pilot implementation, leading to positive health outcomes. For every successful community-based intervention, paying serious attention to critical socio-cultural values of the communities in question is highly paramount [6].

In Ghana, vector control methods are the main malaria control methods used. The provision and use of mosquito nets (LLINs), where the impregnated insecticide keep up to three years and above, and the nets are occasionally washed to keep it clean whiles the insecticide is kept for active use for up to 12 months [11]. It is endorsed by the WHO that all countries and agencies scale up the supply and distribution of mosquito nets, especially for target populations at high-risk areas [11]. A lot of countries malaria prevention programmes have adopted the universal coverage of LLINs supply and distribution, where mass distribution campaigns are conducted between the intervals of two to four years depending on the endemicity levels. Integration of multiple interventions and strategies are the bedrock of global malaria control campaigns, which greatly contributed to the reduction of malaria burden. Most successful countries used global malaria prevention campaign strategies which resulted in the prevention of malaria from such countries and significantly lessened the burden in others [11]. Additionally, the use of chemoprophylaxis for women during pregnancy after quickening, which is delivered through directly observe treatment doses in the form of intermittent preventive treatment in pregnancy to prevent malaria infection in pregnancy thereby reducing the risk of anaemia and other negative birth outcomes [11]. Also, there is another intervention for children where anti-malarial drugs are administered seasonally in the form of chemoprevention for children. These integration and combination of interventions being use to prevention malaria and now with the introduction of the malaria vaccine to add on to the already existing multi strategies for malaria prevention [11].

The malaria vaccine in Ghana is one of the effective malaria prevention interventions, which targets 95% coverage for pilot implementing districts. Kassena Nankana Municipality is among the only two selected implementing areas in Upper East region of Ghana. Since the introduction of the vaccine into the routine Immunization programme in May 2019, the municipality could not cover its annualized target consistently in 2019 and 2020. In the first year of implementation, the municipality covered 48.5% for dose one, 49% for dose two and 40.4% for dose three. During 2020, the municipality covered 43.4% for dose one, 40.5% for dose two and 40.2% for three [12]. Due to poor community entry process, engagement with community leaders, opinion leaders, identifiable groups and all other stakeholders, most people did not accept the vaccine for their children to be vaccinated. Additionally, demand generation, communication, publicity and awareness creation on the importance of the vaccine was poorly done in the communities [12].

In terms of trainings for health staff to be able to administer the vaccine, only few health staff were trained and as a result, the few trained staff could not cover all the communities to vaccinate the eligible children. As a result of this abysmal performance by the municipality, eligible children are left unreached and unvaccinated. This challenge could have been generated by the health service delivery system such as health staff knowledge on vaccination schedules, eligibility criteria, staff attitude, data capture and logistics supply. There could be community factors such as anti-vaccination groups activities, vaccine hesitancy, inadequate knowledge on the malaria vaccine and other social, cultural and religious practices that precipitate vaccine acceptance. Implications for low performance coverage are the accumulation of susceptible unimmunized children which impedes the planned evaluation of the feasibility of administering the scheduled four doses, evaluate the impact on morbidities and mortalities due to malaria and the tracking of the vaccine’s safety when use in the routine vaccination programme to inform policy direction by WHO on the scale up of the vaccine. This study thereby seeks to assess the predictors of the malaria vaccine (RTS,S) uptake among mothers/caregivers with children 6 to 24 months in the Kassena Nankana Municipal in the Upper East Region of Ghana.

## Methods

### Study design and population

The study adopted a cross-sectional design in conducting this study. This study design is referenced about a single point in time for both the exposure and outcome variables.

The rationale for the selection of cross-sectional study design was that, the collection of data was done at a particular point in time. The study population was children age between 6 months to twenty-four months (6 to 24 months) whose mothers/caregivers resided in the municipality. The schedule for the malaria vaccine starts with infants at age 6 months and ends at twenty-four months hence the reason for selecting this age group to determine the factors associated with the uptake.

### Sampling and sample size

Probability sampling method was adopted to select the study participants. At the first stage, a simple random sampling method was used to pick 30 clusters (communities) from the 110 communities in the municipality. Using the balloting method, communities’ names were written on pieces of paper and kept in a container and with vigorous shaking, all the 30 communities were selected. In the second stage, proportion to population size was used to determine the number of children studied in each of the selected clusters. Cumulative population for the selected 30 clusters was determined and, therefore, each cluster’s population was divided by the cumulative population and multiplied by the sample size to determine the study participants in that cluster. Also, in a cluster, sampling of participants was done from the centre of the selected community and followed the direction of the spun pen or pointer, houses in that direction were selected for the survey through the principle of the next nearest household. Children were taken on sequentially until the planned cluster sample size was attained. House-to-house visits and face-to-face interviews were done with mothers/caregivers who had eligible children. In a household where mothers with eligible children were more than one, a simple random sampling method, that is balloting was done to pick one for the study.

The study adopted the formula for sample size determination from Yamane’s formula [13] as indicated below formula:

$$\text{n} = \frac{N}{{1 + N( \propto )^{2} }}$$

where n = sample size, N = study population (8311 target of children 0–24 months), α = margin of error which is 0.05 with significance level of 95%.

Thus, the sample size for the study is calculated as follows:

$$\mathrm{n}=\frac{8311}{1+8311(0.05)2}$$

$$\mathrm{n}=\frac{8311}{21.78}\mathrm{n }= 382$$

Additional 10% non-response rate = (10/100*382) + 382 = 422.

Hence, the sample size of 422 children aged 6–24 months were sampled and studied.

### Data collection and analysis

The study adapted World Health Organization (WHO) cluster survey questionnaire for the data collection. Seven data collectors were trained on the study protocols and the questionnaires. In addition, a pre-test of the tool was done after the training to ensure understanding of the tools. Data were collected electronically by field data collectors using Kobocollect application on android mobile devices (https://www.kobotoolbox.org). After an informed consent and child assent were sought and received from the mothers/caregivers, the selected children’s mothers responded to interviewer-administered questionnaires. Information on malaria vaccine immunization was obtained through review of the children’s vaccination records books and their mothers recall and verbal reports. The mothers were asked to show the interviewer the child health record booklet with immunization dates to authenticate the uptake of malaria vaccine. After the data collection in the field, the administered questionnaire data were downloaded and checked for completeness and cleaned. The extracted data from the KoboCollect server was analysed using the Statistical Package for the Social Sciences (SPSS) version 25.0 [14]. In addition, Stata version 13 [15] was used to calculate the odds ratios. The odds ratios were obtained from binary logistic regression model analysis of the dependent variable RTSS uptake against the independent variables to find the odds of association to determine the predictors of the malaria vaccine uptake with p-values and 95% CI. Descriptive and inferential statistics were computed and presented in frequencies and percentages in tabular forms. Probability values less than or equal to 0.05 was considered statistically significant.

## Results

Socio-demographic characteristics of respondents were captured in data collection and analysed in Table 1 as independent variables, which can directly or indirectly influence malaria vaccine uptake by eligible children. The findings depict that, the mean age of respondents for the study was 27 ± 5 years and average age of children was 15 ± 8 months.

**Table 1:** Demographic characteristics of mothers and children (6–24 months)

Variables	Frequency n (%)
Age of mother/caregiver (years)	
Less than 20	33 (7.8)
20–29	264 (62.6)
30 and above	125 (29.6)
Educational level	
No formal education	84 (19.9)
Primary	60 (14.2)
Middle school/JHS	134 (31.8)
A’Level/SHS	89 (21.1)
Tertiary	55 (13.0)
Marital status	
Married	374 (88.6)
Not married	48 (11.4)
Religion	
Christianity	370 (87.7)
Islam	29 (6.9)
Traditionalist	4 (0.9)
Free thinker	19 (4.5)
Ethnicity	
Kassena	262 (62.1)
Nankana	116 (27.5)
Frafra	28 (6.6)
Others	16 (3.7)
Occupation	
Salary worker	54 (12.8)
Trading/business	175 (41.5)
Farming	125 (29.6)
Artisan	34 (8.1)
Unemployed	34 (8.1)
Relationship with child	
Mother	416 (98.6)
Caregiver	6 (1.4)
Age of child (months)	
Below 12	190 (45.0)
12–23	174 (41.2)
24 months	58 (13.8)
Sex of child	
Male	231 (54.7)
Female	191 (45.3)

Regarding the uptake of RTS,S 94% of the children received full doses of the vaccine and one major reason for not receiving all the doses was the sickness of a child (Table 2).

**Table 2:** Level of uptake of RTS,S

Indicators	Frequency N (%)
Level of uptake	(n = 422)
Poor	26 (6.20)
Good	396 (93.80)
Reasons for child not receiving all doses	(n = 26)
Child was sick	19 (73.08)
Was not around RTS,S implementing district	4 (15.38)
Came for service but not given	1 (3.85)
Mother/Caregiver busy	2 (7.69)
Coverage for RTSS 1, 2 and 3	(n = 364)
RTS,S 1	356 (97.80)
RTS,S 2	356 (97.80)
RTS,S 3	352 (96.70)
Coverage for RTS,S 4	(n = 58)
RTS,S 4	34 (58.62)

There was strong association between health education on the malaria vaccine, given options for acceptance and attitude of health staff during immunization sessions and RTS,S uptake (Table 3).

**Table 3:** Association between health service delivery factors and RTS,S uptake

Variable	RTS,S Uptake		chi^2^	P-value
	Poor	Good		
Educated on malaria vaccine				
Yes	16	372	34.58	**< 0.001**
No	10	24		
Given the option to accept malaria vaccine				
Yes	14	353	26.82	**< 0.001**
No	12	43		
Long waiting time for vaccination in the facility				
Yes	1	15	0.00	0.988
No	25	381		
Experienced Adverse event following immunization				
Yes	5	63	0.01	0.913
No	21	333		
Attitude of health staff during immunization sessions				
Excellent	11	230		
Satisfactory	14	165	1.95	0.163
Disappointing	1	1	8.72	**0.003**

^*^Chi^2^–Chi Square; Bolded p-values depict statistical significance

Community factors that hinder RTS,S vaccine utilization among the study group. There was statistically significant association (p < 0.005) between respondents’ who were ‘somewhat confidence’ in the effectiveness of the RTS,S vaccine. There was also a strong association between those who were ‘Not confident at all’ and still took the RTS,S vaccine (p < 0.001) (Table 4).

**Table 4:** Association between community factors and RTS,S uptake

Variable	RTS.S Uptake		chi^2^	P-value
	Poor	Good		
Heard about negative rumour on RTS,S vaccine				
Yes	3	64		
No	23	332	0.39	0.532
Trust/confidence in the effectiveness of the RTS,S vaccine				
Completely confident	13	311		
Somehow confident	10	84	6.15	0.013*
Not confident at all	3	1	42.91	< 0.001*
Community acceptance of RTS,S vaccine				
Yes	23	253		
No	1	12	0.01	0.935
Don’t know	2	31	0.20	0.651
Community members concern about more vaccine introduction				
Yes	11	177		
No	2	93	2.02	0.155
Don’t know	13	126	1.44	0.230

^*^Chi^2^–Chi Square; Bolded p-values depict statistical significance

Attitude of health staff during immunization sessions being reported by respondents as “disappointing” was a significant predictor of malaria vaccine uptake (P = 0.003). Also, education on malaria vaccine, given the respondents the option to accept the malaria vaccine were all statistically significant predictors of the malaria vaccine uptake (P < 0.001) (Table 5).

**Table 5:** Predictors of RTS,S vaccine uptake

Variable	cOR (95% CI)	P-value	aOR (95% CI)	P-value
Educated on malaria vaccine				
Yes	Ref			
No	9.69 (3.496–25.425)	** < 0.001**	0.27 (0.054–1.324)	0.106
Given the option to accept malaria vaccine				
Yes	Ref			
No	7.04 (2.759–17.476)	** < 0.001**	0.34 (0.073–1.568)	0.166
Attitude of health staff during immunization sessions				
Excellent	Ref			
Satisfactory	1.77 (0.726–4.431)	0.163		
Disappointing	20.91 (0.244–1647.49)	**0.003**	1.51 (0.568–4.036)	0.407
Trust/confidence in the effectiveness of the RTS,S vaccine				
Completely confident	Ref			
Somehow confident	2.85 (1.074–7.293)	**0.013**	0.27 (0.118–0.601)	**0.001**
Not confident at all	71.77 (5.091–3776.875)	**< 0.001**		

Bolded estimates are statistically significant, cOR: crude odd ratio, aOR: adjusted odd ratio, 95% CI: 95% confident inferential, p <  = 0.05 considered statistically significant

Caregivers who had not received education on the malaria vaccine were 9.69 times more likely to receive the vaccine compared to those who have received education [cOR = 9.69 (CI 3.496–25.425), p < 0.001]. Again, those who were not given the option to accept the malaria vaccine were 7.04 times more likely to receive the vaccine compared to those who were given the option for the vaccine uptake education [cOR = 7.04 (2.759–17.476), p < 0.001].

Surprisingly, caregivers who rated the attitude of health staff during immunization were 20.91 times more likely to take the vaccine compared to those who rated them excellent [cOR = 20.91 (0.244–1647.49), p = 0.003].

## Discussion

### Level of uptake of RTS,S

This study assessed the level of uptake of RTS,S vaccine performance and elements related with it in children 6 months to 24 months old in Kassena Nankana Municipal of the Upper East Region. When the children immunization statuses were confirmed using vaccination cards and mothers recall method, it came to light through the study findings that, fully immunized coverage was high (94%) among the study participants. This figure is in line with WHO’s Global Vaccine Action Plan which proposes that countries attain about 90 percent and districts attain about 80 percent fully immunized children by the year 2020 [16]. The findings from this study showed a comparatively similar results to a study in Sunyani [16] in the Bono Region of Ghana, which indicated that uptake of RTS,S first dose was 94.1 percent. Nonetheless, this figure declined to 90.6 percent for RTS,S second dose, and 78.1 percent for RTS,S third dose. Therefore, this high immunization coverage in this study implies that there was herd immunity among children in the district and, therefore, the risk of vaccine-preventable diseases like malaria is expected to be low in counts and severity.

Although, this finding clearly indicates that the municipality may not have high number of unimmunized children as the administrative coverage highlights, there may be unimmunized children out there that the municipality needs to strategically trace and immunize them to reach every child in the catchment area to achieve optimal immunization coverage. The entire municipality has a lot of rural setting with 80 percent of its facilities as Community-based Health Planning and Services (CHPS), therefore, through the activities of CHPS (home visits, defaulter tracing, and vaccination, among others), unimmunized children can easily be traced and immunized. Immunization performance based on children vaccination cards and mothers’ memory recall for the RTS,S vaccine was high as presented in the results section in this write up, these findings were in contrast to the municipal vaccine administration rate as it had been recording low vaccination coverage per its targets as presented in Upper East Regional Health Directorate Annual Report, 2020. In the first year of implementation (2019), the municipality covered 48.5%, 49% and 40.4% for first, second and third dose respectively and in the second year (2020), the municipality covered 43.4%, 40.5% and 40.2% for first, second and third dose, respectively [12]. These discrepancies between the municipality’s low administrative coverages and these high study findings coverages are unexplained because the study did not cover immunization service providers’ perspectives on the low administrative coverage. However, these findings can suggest that staff with inadequate knowledge in charge in handling immunization data and inadequate supervision and monitoring may contribute to low administrative coverage. Appropriate screening of vaccination status might not have been done by the health staff when mothers/caretakers came to health facilities with their children for preventive and curative services may also contribute to low administrative coverage. Another possible explanation can be attributed to poor data management including erroneous population indicators. Further statistical analysis using odds ratio calculation and chi-square to determine which variables relate significant with the uptake level of the malaria vaccine, showed that; ‘education on malaria vaccine’ (P < 0.001) and ‘given option to caregivers to accept malaria vaccine’ (P < 0.001) had significant associations as health service factors and the uptake of the vaccine. Community factors that showed statistically significant association with malaria vaccine uptake is confidence in the effectiveness of the vaccine (P < 0.005). However, attitude of health staff during immunization sessions being reported by respondents as “disappointing” was a significant predictor of malaria vaccine uptake (P = 0.003). These associations can provide cues and it is a wake-up call for public health workers in the municipality to strengthen education on vaccines to communities, especially caregivers/mothers. Good working relationship and engagement with their clients as well as improved inter-personal communication should be the hallmark for staff in achieving the set goals of reaching every eligible child in the municipality with the required vaccines including malaria vaccine. Significantly caregivers’ education to keep the immunization cards as a source of documentation and records to track children immunization to its completeness should be prioritized. Also, education on the second year of life immunization should be prioritized to lessen the risk of malaria episodes in the municipality.

### Health service delivery factors associated with RTS,S uptake

Factors affecting immunization services are often obstacles within the health service and caregivers’ factors. Among the study participants there were high acceptance rate of the malaria vaccine because most of the respondents were educated by the health staff on the vaccine. Majority of the respondents said their children did not experience any adverse events following immunization, which could have affected the vaccine uptake. Additionally, most of the respondents were satisfied with the attitude of the health staff. A majority of the respondents who missed the full uptake were as a result of their children been sick or were not around RTS,S implementation catchment areas. These findings were in contrast with the assertion of Van Den Berg et al. [6] on the various reasons giving by caregivers on why they could not complete their ward’s immunization ranges from obstacles such as inconvenient timing for immunization services, mother’s too busy and long waiting time; inadequate information on place of vaccination unknown and lack of motivation like postponing of immunization services. Additionally, no respondent mentioned poor health staff attitude, inadequate vaccination skills that cause adverse event following immunization, unapproved charges by health staff, unplanned and lack of communication on changes in schedules and migrations to contribute to none completion of immunization. The study did not reveal similar findings with Ballou’s study [17] that indicated that caregivers paid for immunization services, that notwithstanding, lack of information on immunization and time factor were supported by these findings. The findings once again showed that people were reachable with health services even though few nomads and seasonal migrants were encountered and strategic approach is needed to reach out to all eligible children for immunization. This will buttress the assertion by Dimala et al. [18] that immunization coverage is affected by several factors and to achieve the optimal coverage level, systems thinking and strengthening is imperative. As it was stated by Abdulkadir et al. [19] that factors affecting immunizations are frequently due to supposed and real deficits within the health sector such as inadequate information on immunization services. This is often the main hindrance to attaining full immunization of children and women. Mostly, they might not know the places and date or time for the vaccination, this study proved otherwise as the respondents were well aware and educated on the immunization services. The reasons that hindered caregivers from completing their children’s immunization and created gaps in immunization services can be categorized under health system factors and caregivers’ factors, therefore, for successful implementation and sustainability of EPI services, identification and addressing of these gaps is paramount [20]. The findings support the above assertion and if steps are taking to address identified gaps, immunization services will be improved.

### Community factors influencing RTS,S uptake

Access to immunization services was good as most of the respondents alluded to the fact that they spend less time in accessing immunization services. Factors affecting immunization services are often obstacles within the health service and caregivers’ factors. However, community factors such as negative rumours, vaccine hesitancy, religious and cultural disbelieve in orthodox medicines including vaccines most often than not affect vaccine uptake. In the case of this study findings, the vaccine acceptance rate was high among the study participants. The findings once again buttress Mukungwa’s pronouncement [21] that mothers and care givers awareness of immunization schedule and consistency of immunization schedule delivery increase the probability of children being fully immunized at the appropriate age. However, education level of the respondents did not have significant impact on the uptake level of the RTS,S vaccine as it was noted by [22] that people are well placed in the social class in terms of education and jobs are likely to use health services more than those perceived to be in the lower social class. Moreover, individuals’ cultural belief also impacts the level of use of services such as immunization. This study findings revealed similar output by Dimala et al. [18], who indicated that the acceptance and uptake of the RTS,S vaccine may be improved if caregivers’ perceptions about vaccines and their importance are adequately informed and supported through engagement and education. A study on the uptake of RTS,S vaccine conducted in Sunyani by Tabiri et al. [16], found that Uptake of 1st and 2nd doses met WHO’s target, however, the subsequent doses were low as a result of increasing negative perception in communities that injectable vaccines are becoming much more for children, which negatively affects uptake. This study found similar high uptake with kept dropping out as a result of missed opportunities due to vaccine shortages, competing health programmes and interventions. Health workers play very important roles in working closely with their communities as the respondents constantly cited health staff as their dependable sources of health information on vaccination, therefore it is imperative for health staff to educate and mobilize communities’ support for vaccination and to use immunization services. This necessitates health staff and other people who have tried and tested the system to keep caregivers informed of places and time that they need to bring children for vaccination [23].

According to Van Den Berg et al. [6], integrating community values in vaccination exercise will help address real challenges in most trials and pilot implementation of intervention leading to positive health outcomes. As the communities begin to show concerns about more vaccine’s introductions, there should be frantic efforts by health authorities to systematically engage all stakeholders including community leaderships, opinion leaders and all who matter to build consensus and this will help to dispel most of the negative rumours about the safety of vaccines as also indicated by Meñaca et al. [24], in a study which identified that number of challenges such as the hesitancy, rumours and misinformation by some people about RTS,S vaccine could be addressed through planned communications strategy and simple messages. Adopting the recommendations of Dimala et al. [18] that operational implementation of RTS,S vaccine necessitates vigilant thoughtfulness of the social, religious and cultural perspective of each community through community engagement and involvement and the establishment of adequate health information system in an acceptable form through consistent communication channels should be the way to go. In essence, this study findings and what is known in the existing literatures point out to the fact that for a pilot district to achieve high uptake of malaria vaccine, there is the need to harmonize the health service goals and actively engage the communities to address their issues and concerns on the growing numbers of injections among childhood vaccinations. However, some limitations of this study were that, recall bias from mothers/caregivers’ verbal reports as some might have not recall past events correctly. This is due to the study design used, but this did not affect the outcome of the study so much.

## Conclusion

Generally, malaria vaccine uptake among children was high in the Kassena Nankana Municipality, which can contribute to the level of protection against the risk of prevalence of malaria episodes among children leading to preventable deaths. However, full uptake of the vaccine among the children by age two, especially the fourth dose declined as the children aged which was an indication of high drop-out rate.

The findings of this study have health policy implications for the health system in Ghana. The gaps found as predictors for the caregivers on routine immunization services may lead to low use of the health information, planning and decision-making. The low administrative immunization coverage data may also lead to funding implications and supply of vaccines which can be negatively affected during outbreaks or emergencies. The study also buttresses and confirms most of the literature assertions on Expanded Programme on Immunization, however, this finding can provide an opportunity to address the existing gaps and improve the overall health system.

## Data Availability

Data can be obtained from the corresponding author on reasonable request.
